# SARS-CoV-2-related pneumonia cases in pneumonia picture in Russia in March-May 2020: Secondary bacterial pneumonia and viral co-infections

**DOI:** 10.7189/jogh.10.-020504

**Published:** 2020-12

**Authors:** Konstantin S Sharov

**Affiliations:** Koltzov Institute of Developmental Biology of Russian Academy of Sciences, Moscow, Russia

## Abstract

**Background:**

We are communicating the results of investigating statistics on SARS-CoV-2-related pneumonias in Russia: percentage, mortality, cases with other viral agents, cases accompanied by secondary bacterial pneumonias, age breakdown, clinical course and outcome.

**Methods:**

We studied two sampling sets (Set 1 and Set 2). Set 1 consisted of results of testing 3382 assays of out-patients and hospital patients (5-88 years old) with community-acquired and hospital-acquired pneumonia of yet undetermined aetiology. Set 2 contained results of 1204 assays of hospital patients (12-94 years old) with pneumonia and COVID-19 already diagnosed by molecular biological techniques in test laboratories. The results were collected in twelve Russian cities/provinces in time range 2 March – 5 May 2020. Assays were analysed for 10 bacterial, 15 viral, 2 fungal and 2 parasitic aetiological agents.

**Results:**

In Set 1, 4.35% of total pneumonia cases were related to SARS-CoV-2, with substantially larger proportion (18.75%) of deaths of pneumonia with COVID-19 diagnosed. However, studying Set 2, we revealed that 52.82% patients in it were also positive for different typical and atypical aetiological agents usually causing pneumonia. 433 COVID-19 patients (35.96%) were tested positive for various bacterial aetiological agents, with *Streptococcus pneumoniae, Staphylococcus aureus* and *Haemophilus influenzae* infections accounting for the majority of secondary pneumonia cases.

**Conclusions:**

SARS-CoV-2, a low-pathogenic virus itself, becomes exceptionally dangerous if secondary bacterial pneumonia attacks a COVID-19 patient as a complication. An essential part of the severest complications and mortality associated with COVID-19 in Russia in March-May 2020, may be attributed to secondary bacterial pneumonia and to a much less extent viral co-infections. The problem of hospital-acquired bacterial infection is exceptionally urgent in treating SARS-CoV-2 patients. The risk of secondary bacterial pneumonia and its further complications, should be given very serious attention in combating SARS-CoV-2.

One of the most serious complications of COVID-19 and the main cause of acute respiratory distress syndrome and respiratory failure that, in turn, often result in death of a patient, is atypical pneumonia [1,2]. However, the symptomatic clinical picture of pneumonia related to SARS-CoV-2 in most cases resembles pneumonia caused by many other viral agents [2-5]. Conversely, it has been observed that the considerable number of serious patients with viral pneumonia hospitalised during last several months, exhibited clinical symptoms and/or computer tomography (CT) images very similar or even identical to those that one can usually see in COVID-19 positively tested patients, but no SARS-CoV-2 was detected in respiratory samples [3,6-10].

This may potentially lead to several clinical confusions that would further distort the statistics on COVID-19 and prevent us from evaluating its real hazard in serious cases [11,12]. First, there may be underestimating the role of SARS-CoV-2 in the overall pneumonia picture in Russia because of possible insufficient sensitivity of the testing procedures or high percentage of false-negative results. Similar situations were faced and already reported for different countries [13-15]. Second, one can exaggerate SARS-CoV-2 role in some specific cases, including fatalities, by wrongly ascribing a death of pneumonia or its complications solely to COVID-19, whereas different aetiological agents might be present in addition to SARS-CoV-2 [16]. Finally, there is still no clear understanding of the role of secondary pneumonias (eg, bacterial or fungal) and co-infections with other respiratory viruses in the totality of COVID-19 patients.

In our research, we study clinical course and outcomes of COVID-19-related pneumonia in Russia in March-May 2020, but it may be significant for other countries, where similar questions arose in regard to SARS-CoV-2 and other coronaviruses [17-20], and therefore, for the global health care system. Specifically, we are communicating the following results: percentage, mortality, clinical course, outcomes, cases with other viral agents, and cases accompanied by secondary bacterial pneumonias.

## Hypothesis

Our hypothesis is that SARS-CoV-2, though not very dangerous pathogen itself, becomes very hazardous in cases of secondary bacterial pneumonia associated with it, in terms of both severe clinical course and unfavourable prognosis. Further, the hypothesis assumes that the chief part of mortality ascribed to COVID-19, is caused by secondary bacterial pneumonia, both community-acquired and nosocomial.

## METHODS

### Objects

We studied two sampling sets (Set 1 and Set 2). Set 1 consisted of 6 × 3382 assays of out-patients and hospital in-patients (5-88 years old) with community-acquired and hospital-acquired pneumonia of yet undetermined aetiology: 3382 nasopharyngeal swabs taken thrice from every patient, 3382 sputum or bronchoalveolar lavage (BAL) samples, 3382 blood samples and 3382 urine samples. Swabs were taken three times with the interval of approximately 12-24 hours. Set 2 contained 3 × [2 × 1204] respiratory assays and 3 × 1204 sputum/BAL samples of hospital patients (12-94 years old) with pneumonia and COVID-19 already diagnosed by molecular biological techniques. Nasopharyngeal swabs were taken twice with the interval of nearly 12 hours to reduce false-negative results occurrence for each of the three global intakes. These three global intakes of swabs and sputum/BAL were carried out in hospitals according to the following scheme: 1) at the moment of a patient hospitalisation; 2) on day 4 of in-hospital treatment or when the clinical picture deteriorated; and 3) on day 10 of hospitalisation or when the clinical picture severely deteriorated. The presence of such triple intake allowed us to analyse a posteriori the development of hospital-acquired viral and bacterial complications of SARS-CoV-2 pneumonia. Sets 1 and 2 were non-overlapping. The study size was limited by time interval, test laboratory capacities and rate of providing clinical information by clinicians. The size of Set 2 (1,204) can be estimated as some 5% of all pneumonia cases with COVID-19 diagnosed by 5 May 2020 in Russia. Therefore, its investigation may represent a significant contribution to understanding the total situation with COVID-19-related pneumonias in Russia in March-May 2020.

### Sensitivity

Multiple taking the swabs was carried out to minimise occurrence of false-negative results. Maximal values of DNA/RNA genome equivalents per reaction, obtained in three (Set 1) and two (Set 2) consecutive measurements: N = max{N_i_},i = 1,2, (3) were used. For all viral aetiological agents, amounts of less than 50 DNA/RNA genome equivalents per reaction were considered as the absence of a virus in a human organism, more than 50 as its presence. This approach fixes a twice-stricter threshold than the common threshold of 100 RNA genome equivalents per reaction used in the official programme of mass screening for SARS-CoV-2 in Russia.

### Clinical data

All clinical data used in the current study were anonymised. Information on age, gender, results of analyses, clinical picture and outcome was collected in twenty-four Russian hospitals transformed to COVID-19 infirmaries, twenty-two outpatients’ clinics, eight ambulance centres, eight non-commercial test laboratories and medical centres. The assays of both sets were collected in twelve Russian cities/provinces (Moscow, Moscow region, St Petersburg, Nizhny Novgorod, Murmansk, Syktyvkar, Khabarovsk, Krasnodarsky Kray, Krasnoyarsk, Tula, Vladivostok and Volgograd) in time range 2 March – 5 May 2020. These regions most suffered from COVID-19 pandemic in Russia. Both in-patients and out-patients with pneumonia were included in the samplings (Sets 1 and 2). Community-acquired pneumonia and hospital-acquired pneumonia were distinguished.

### Testing procedures

Pneumonia was diagnosed by hospital medical personnel on the basis of x-rays CT screening. Initial (primary) testing procedures were as follows. Viral aetiological agents were identified in nasopharyngeal swab samples by testing laboratory personnel using molecular biological methods. Bacterial aetiological agents were revealed in sputum samples by cultural (inoculation) techniques with a following biochemical identification and biochemical tests and/or antigen detection in blood and/or urine samples. Fungal aetiological agents were identified in sputum by histological analysis. Protozoa aetiological agents were detected in blood films by microscopy. All aetiological agents were detected by qualitative approach (present / not present). Secondary procedures applied by us were surveying hospital and test laboratory personnel, sorting and categorising the data of samplings testing, statistical treatment (where appropriate) and necessary calculations. OriginLab Origin 8.1 (OriginLab Corporation, Boston, MA, USA), .NET Framework 4.5 Silverlight tools, MS Visual Studio 2010 (Microsoft Inc, Seattle, WA, USA) software packages were used for computations and plotting.

### Statistical analysis

Correlation and regression analyses were performed. OriginLab Origin 8.1 and StatSoft Statistica 10 (Tibco, Palo Alto, CA, USA) were used for statistical treatment.

### Ethical guidelines and approval

Reporting of the study conforms to broad EQUATOR [21] and STROBE-NI cohort studies [22,23] guidelines. In hospitals and clinics involved in treating the patients, a written informed consent has been taken from every patient concerned that he/she gives a clear permission to use their data for scientific research and publications. All written informed consents duly signed are kept in the hospitals, clinics, ambulance and medical centres. The Ethical Committee of Koltzov Institute of Developmental Biology of Russian Academy of Sciences regarded the current study ethically appropriate and exempt from human subjects review, as no clinical trials were performed, the authors were not personally involved in collecting the clinical data and hence do not possess any information that might identify the patients.

## RESULTS AND DISCUSSION

### Pneumonias of different aetiology in Set 1

**Figure 1:**
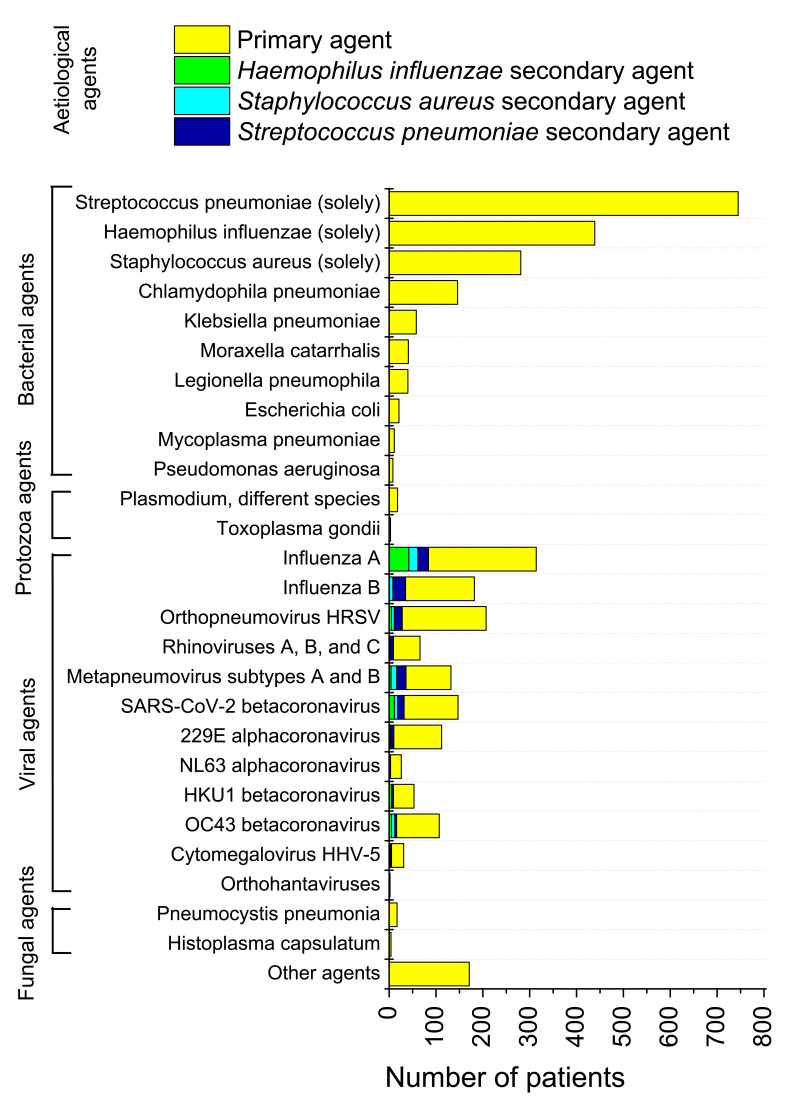
Results of testing the broad sampling of pneumonia patients (Set 1) for different pneumonia aetiological agents. Viral agents were detected in swab samples. For Orthohantavirus, hantavirus pulmonary syndrome was detected. For different detected species of *Plasmodium* (*P. falciparum, P. malariae, P. ovale, P. vivax*) pulmonary oedema was observed. Protozoa-caused pneumonias were detected for some persons, which recently returned from epidemiologically risky areas of Africa, South Asia and Latin America. *Plasmodium* was detected in blood films by microscopy. Fungal agents were revealed mainly in immunocompromised persons (sputum, histology). These cases were also included in the overall pneumonia picture. Cytomegalovirus (swabs) and *Escherichia coli* (K1 antigen detection in blood) that were deemed to have caused pneumonias, were found only in the children and adolescents group (5-17 years). For viral pneumonia cases, tests were also made for secondary bacterial aetiological agents, with *Streptococcus pneumonia* (antigen detection in urine), *Staphylococcus aureus* (sputum/BAL, Gram cytobacterioscopy + cultural inoculation) *and Haemophilus influenza* of serotypes *a, b, c, d, e* and *f* (*IgG* antibodies to PRP antigen detection in blood + sputum/BAL cultural inoculation) infections being mainly detected (blue, cyan and green subcolumns, correspondingly). Secondary bacterial pneumonia statistics include both nosocomial and community-acquired cases. In 171 cases (5.06%), no common causative agent was identified.

On 9 April 2020, the Russian Official Headquarters on Control and Monitoring of Coronavirus Situation announced that the majority of pneumonia cases in Moscow were caused by COVID-19 [24]. However, our study of Set 1 (3 × 3382 swab samples) demonstrated that SARS-CoV-2 accounted for a small amount of pneumonia cases in the overall pneumonia picture in Russia. We found that only 147 of 3382 pneumonia cases (4.35%) were SARS-CoV-2 positive. This value was the maximum of [3.90%; 4.29%; 4.35%]. False-negative results received in the study, may be accounted for by features of the swab intakes, not precision of testing techniques, completely in line with works [25-28]. The least age of pneumonia patients with SARS-CoV-2 in Set 1 was 16 years old (swab testing+CT). Results of testing for viral, bacterial, fungal and protozoa aetiological agents causing pneumonia, are presented in **Figure 1**. In 92 cases (2.75%) “grey zone” questionable results were obtained. Recurring tests of the same samples allowed to classify almost all of these results properly.

The majority of pneumonia patients, 1790 of 3382 persons (52.92%) had bacterial typical and atypical pneumonia. A little lesser proportion, 1379 of 3382 samples (40.77%) were tested positive for different acute respiratory infection (ARI) viral aetiological agents, with Influenza A giving maximal contribution of 314 samples (9.28%) to the total pneumonia picture. That amount is more than twice as large in comparison with the number of SARS-CoV-2-related pneumonias. Four common coronaviruses in seasonal circulation (HCoV-HKU1, HCoV-OC43, HCoV-229E, and HCoV-NL63) mutually accounted for 298 cases of pneumonia, which is also considerably higher than the number of SARS-CoV-2 related pneumonias in Set 1.

### Mortality in Set 1

**Figure 2:**
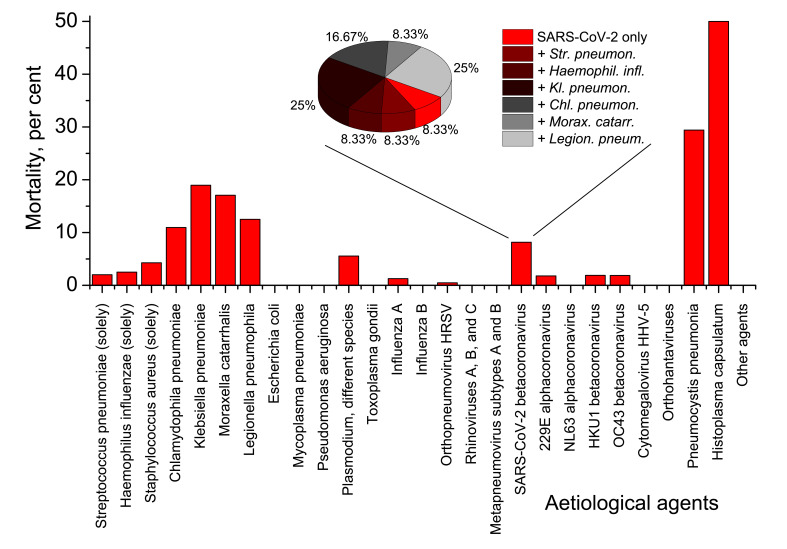
In-group mortality rates in Set 1. Mortality of SARS-CoV-2-related pneumonia are further subdivided into different groups in the inset. 91.67% of lethal cases associated with COVID-19, were also related to different secondary bacterial pneumonias.

Despite low proportion of SARS-CoV-2-related cases in the whole pneumonia picture, mortality from pneumonia and its further complications related to SARS-CoV-2, was considerably higher than average mortality of pneumonia and its complications in the broad sampling (**Figure 2**). Of complete Set 1, 64 fatalities were registered, and 12 fatal cases were in the group of 147 SARS-CoV-2 positive persons. That gives 1.89% mortality rate for the broad pneumonia sampling, and 8.16% mortality rate for SARS-CoV-2-related pneumonia subsampling. Therefore, of all deaths due to pneumonia and its complications in Set 1, SARS-CoV-2-related cases accounted for 12/64×100%=18.5%. Usual case fatality rate of pneumonia of different aetiology was some 3%-5% in Russia for 2013-2019 [29].

The overwhelming majority of mortalities in Set 1, viz. 52 of 64 cases (81.25%) were directly or indirectly associated with primary or secondary bacterial pneumonias. 7 fatalities (10.94%) were of immunosuppressed patients with fungal pneumonias. One death case (0.68%) of viral Influenza A pneumonia, that was complicated by pulmonary oedema caused by *Plasmodium falciparum*, was observed. Only four lethal cases were connected with viral pneumonias not complicated by any secondary cases or co-infections: two were deemed to be caused by Influenza A (1.36%), one by human respiratory syncytial virus (0.68%), and one by SARS-CoV-2 (0.68%). Importantly, only one death of twelve registered for SARS-CoV-2-related pneumonia, was not associated with secondary bacterial infection (**Figure 2****,** inset).

### Secondary bacterial pneumonias in Set 1

We observed that for persons tested positive for SARS-CoV-2 and different viral agents in Set 1, the proportion of secondary bacterial pneumonias was high, as in China [30-33]. These secondary pneumonias were nosocomial or community-acquired. Of 147 SARS-CoV-2-related pneumonias, 61 cases (41.50%) were positive for different bacterial agents, with *Streptococcus pneumoniae* accounted for 32 cases of secondary bacterial pneumonias, *Staphylococcus aureus* for 18, and *Haemophilus influenzae* for 11 cases. Other pneumonia bacterial aetiological agents accounted for a lesser amount of secondary infections in viral interstitial pneumonia cases revealed in Set 1. In comparison, for pneumonias associated with Influenza A, 59.55% cases were with secondary bacterial pneumonias, Influenza B 23.63%, respiratory syncytial virus 21.26%, and metapneumoviruses 42.42%. For other coronaviruses, a lesser percentage with bacterial pneumonias was observed in comparison with SARS-CoV-2, viz. 80 of 298 cases (26.85%) in total.

### Test results for different pneumonia aetiological agents in Set 2

**Figure 3:**
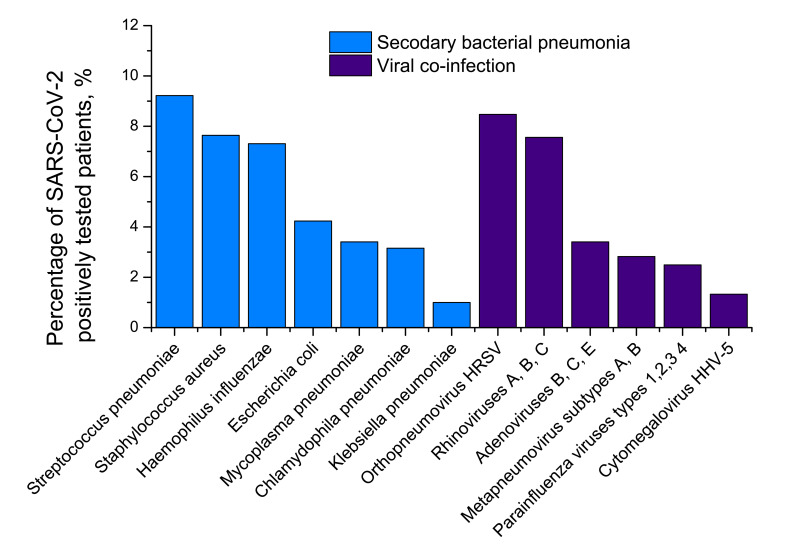
Results of testing pneumonia patients with SARS-CoV-2 (Set 2) for different viral and bacterial aetiological agents causing pneumonia. If in any of the three samples (see Methods section for a detailed explanation) bacterial agents were identified by cultural inoculation or another techniques, we counted the case in question as complicated by bacterial pneumonia. Percentage of COVID-19 patients with secondary bacterial pneumonia and different viral co-infections is shown. *Escherichia coli* secondary bacterial pneumonia was observed for 12-17 age group. Detection. *Streptococcus pneumoniae*: urine, antigen; *Staphylococcus aureus*: sputum/BAL, Gram cytobacterioscopy + cultural inoculation; *Haemophilus influenzae* of serotypes *a, b, c, d, e* and *f*: blood, IgG antibodies to PRP antigen detection, sputum/BAL, cultural inoculation; *Escherichia coli:* blood, K1 antigen; *Mycoplasma pneumoniae*: blood, *IgA+IgM* antibodies; swabs, multiplex RT-PCR for *Mycoplasma* DNA detection; *Chlamydophila pneumoniae*: blood, *IgM* antibodies; swabs, multiplex RT-PCR for DNA detection; sputum/BAL, culture inoculation; *Klebsiella pneumoniae*: sputum/BAL, spectrophotometry assay for detection of *Kl. pneum.* carbapenemase KPC+RT-PCR for bla-KPC gene; sputum/BAL, cultural inoculation + detection of indole, ornithine decarboxylase, acetone (Voges-Proskauer reaction), o-nitrophenyl-β-D-galactopyranoside synthesis malonate utilization in a broth culture; *Legionela pneumophila*: urine, antigen, specific buffered charcoal yeast extract (BCYE) alpha culture inoculation with cysteine and Fe_4_(P_2_O_7_)_3_; *Moraxella catarrhalis*: swabs, multiplex RT-PCR for DNA detection, sputum, culture inoculation; *Pseudomonas aeruginosa*: blood, IgG antibodies; sputum/BAL, culture inoculation in trypticase soy agar; all virus agents: multiplex RT-PCR technique.

Set 2 consisting of respiratory swabs, blood/urine and sputum/BAL samples of hospital patients already tested positive for SARS-CoV-2, was investigated for different viral and bacterial aetiological agents. The results are presented in **Figure 3****.**

### Age breakdown of complicated SARS-CoV-2 pneumonia

**Figure 4:**
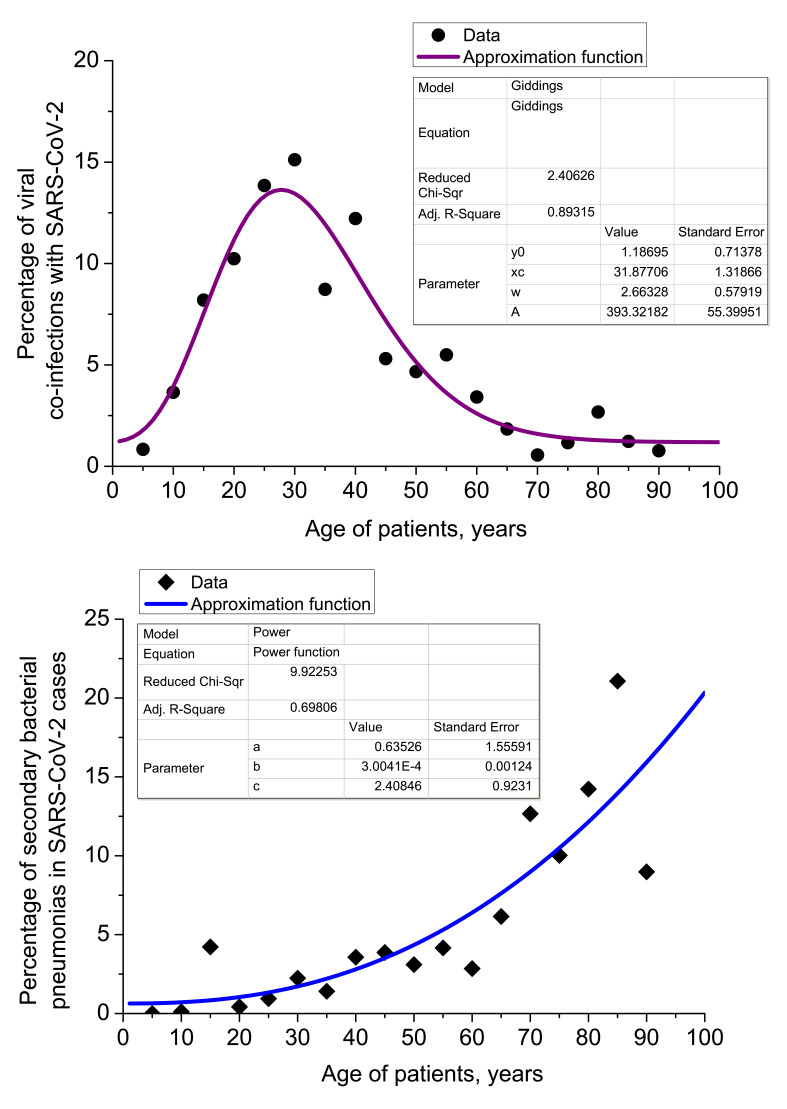
Regression analysis of viral co-infections (left) and secondary bacterial pneumonias (right) in Set 2. Viral co-infection dependency on age is fitted with asymmetric Giddings peak function introduced by J. Calvin Giddings [34-36] (explanation in the **Online Supplementary Document**). Secondary bacterial pneumonia percentage grows with age and may be satisfactorily fitted with a power function: *P_second. act. pneum._ = a + bt^c^.* Factors of regression quality (*χ_red_*^2^, *R_adj_*^2^), coefficients and errors of regression are provided in the figure (inset tables).

The age breakdown of 636 COVID-19 patients with co-infections or secondary infections, is provided in **Figure 4**. While secondary bacterial pneumonia complications become more common with age, we found that viral co-infection rate maximum corresponded with the most active period of life (18-29 and 30-45 years). Statistical regression analysis of dependency of viral co-infections with SARS-CoV-2 and secondary bacterial pneumonias is also provided in **Figure 4**.

Correlation analysis of viral co-infections and secondary bacterial pneumonias in Set 2 gives values of Pearson correlation coefficient *C*_corr._ = -0.52905 at significance level *P* = 0.02397. It means that viral co-infections with SARS-CoV-2 and cases of secondary bacterial pneumonia are mostly uncorrelated in terms of their dependency on age, but for some age groups little counter-correlation is observed.

### Secondary pneumonias and co-infections in Set 2

**Figure 5:**
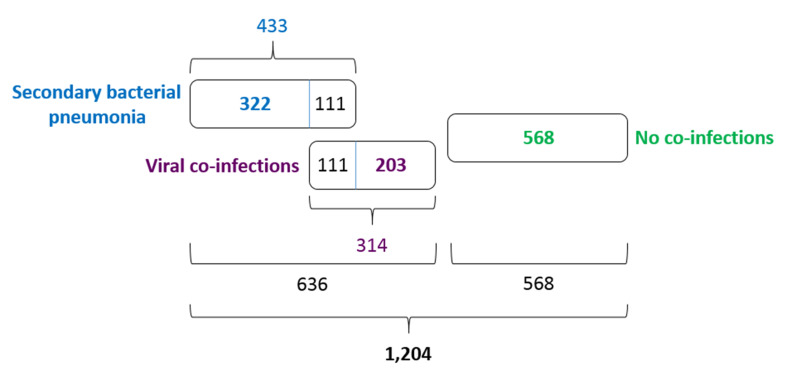
The structure of Set 2. The proportion of secondary bacterial pneumonias, viral co-infections and SARS-CoV-2 pneumonias not complicated by different aetiological agents.

Of 1204 patients with COVID-19, 433 (35.96%) were tested positive for different bacterial aetiological agents. It is likely that these 433 cases should be understood as secondary bacterial pneumonia cases caused by viral pneumonia as complications of COVID-19 disease, and not just as bacterial co-infections. The pneumonia cases were both community-acquired and nosocomial. However, we did not have CT images to make any positive conclusions about the stage and severity of the bacterial pneumonias concerned. Further, 314 COVID-19 patients (26.08%) were tested positive for different viral aetiological agents, with human respiratory syncytial virus and rhinoviruses A, B, C detected to be the most common. The viral co-infections and secondary bacterial pneumonia cases were partly overlapped (**Figure 5**). They led to 111 mutual cases (9.22%) of viral co-infection AND bacterial secondary pneumonia. In total, 636 of 1204 COVID-19 patients (52.82%) demonstrated a viral co-infection OR secondary bacterial infection.

### Community-acquired and hospital-acquired pneumonia

**Figure 6:**
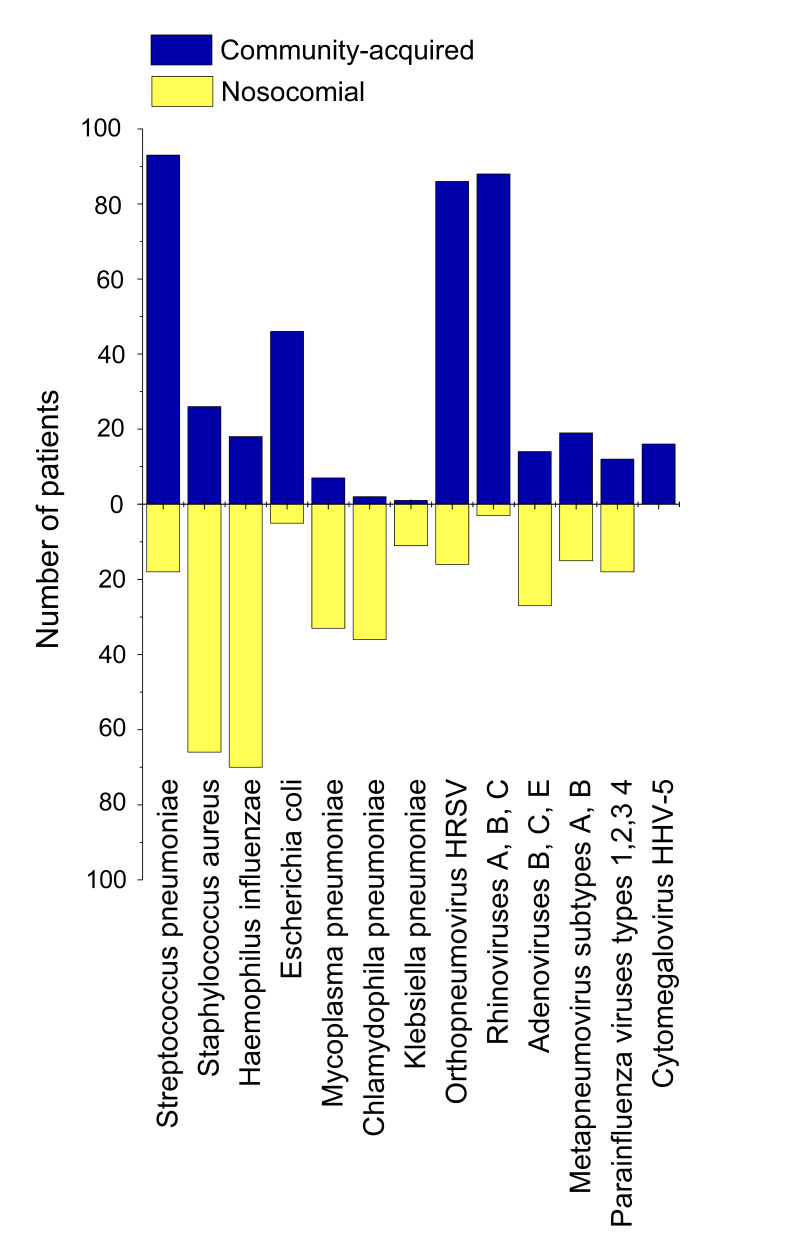
Proportion of community-acquired and hospital-acquired secondary bacterial pneumonias and viral co-infections in pneumonia patients tested positive for SARS-CoV-2 (Set 2).

**Figure 6** demonstrates proportion of community-acquired and hospital-acquired pneumonia with regard to different secondary bacterial and viral aetiological agents complicating SARS-CoV-2 pneumonia cases in Set 2. We did not isolate a separate group of health care-associated pneumonia in our research. All health care-associated cases were treated as hospital-acquired ones, if bacterial agents were detected after 48 hours of hospital stay, and as community-acquired cases otherwise. While *Streptococcus pneumoniae* remained the main causative agent of secondary pneumonia acquired out of hospital, *Staphylococcus aureus* and *Haemophilus influenzae* were revealed to cause much more cases of hospital-acquired pneumonia. Secondary pneumonias related to *Escherichia coli* in SARS-CoV-2 patients of 12-17 years old had been brought about almost completely in community, whereas *Mycoplasma pneumonia* (atypical), *Chlamydophila pneumonia* (atypical) and *Klebsiella pneumonia* (Gram-negative) had appeared almost completely in hospital environment, as they were revealed in sputum/BAL samples on the second or third intake (see Methods for detailed description). In total, 239 of 433 secondary pneumonias (55.20%) were contracted in hospital. This large proportion of nosocomial secondary bacterial pneumonia indicates that the problem of hospital-acquired complications in SARS-CoV-2 patients is very urgent in Russia. All steps should be taken to reduce the number of hospital-acquired infections that are very dangerous for COVID-19-related pneumonia patients, especially those of risk groups.

Though there is no such pressing a problem for viral nosocomial co-infections, and their amount is significantly less than the number of bacterial in-hospital infections (25.16% vs 55.20%), a matter of serious concern is still present. In Set 2, SARS-CoV-2 infection was already proven to be present at the moment of hospitalisation, and 25.16% of patients contracted other co-infecting viruses in hospital environment in addition to SARS-CoV-2, with adenoviruses causing the largest proportion of co-infection cases. But we have very little knowledge, if any, about whether a person from another sampling (outside Set 2) can or cannot contract SARS-CoV-2 in hospital, if he/she is hospitalised with other ARI diseases. In Russia, since 20 April 2020, enormous number of people had been hospitalised with ARI symptoms, and COVID-19 diagnosis was not confirmed afterwards [37]. Most of such persons were discharged after 1-2 days of staying in COVID-19 infirmaries where they had been in a close contact with people tested positive for COVID-19. Therefore, they formed and still continue to potentially form an epidemiologically hazardous group, because chances of their contracting SARS-CoV-2 in hospital might be high.

Currently, much more data are required on mutual viral co-infection with SARS-CoV-2. Eg,, we do not know if some viral infections may facilitate SARS-CoV-2 contraction. Analysing Set 2, we did not reveal any SARS-CoV-2 co-infections with flu viruses and other coronaviruses. This may be potentially explained by their similar stereometric configuration and/or molecular mechanisms of attachment to cellular surface in a human organism, that may result in their competitive exclusion. However, the rate of viral co-infection of SARS-CoV-2 and different ARI viruses other than flu viruses and coronaviruses, is still considerable (26.08%) in Set 2.

To sum up, strict measures should be taken in COVID-19 infirmaries and wards to prevent patients from reciprocal infecting, both viral and bacterial. Mutual re-infection in hospital environment is dangerous, as it is an additional path of SARS-CoV-2 spread in risk groups of population. Critically ill people, aged persons, immunosuppressed individuals, people with many comorbidities are hospitalised in a prioritised manner and later they stay in hospitals longer than other COVID-19 patients. They are likely to be the primary target for secondary bacterial infections and viral co-infections.

### Clinical course, outcomes and mortality in Set 2

**Figure 7:**
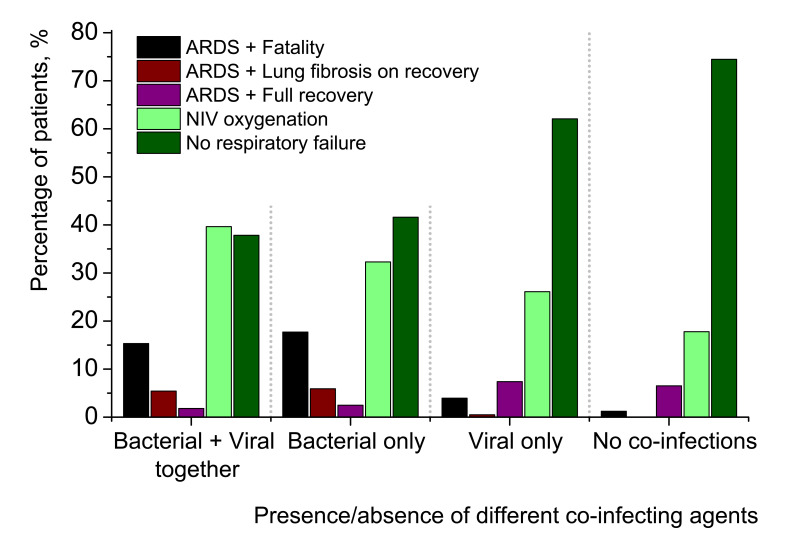
Clinical course and outcomes for 1204 pneumonia patients tested positive for SARS-CoV-2 (Set 2). Oxygenation through Non-invasive ventilation (NIV) was applied for patients with slight degrees of respiratory failure of type 1 (hypoxemic respiratory failure). Acute respiratory distress syndrome (ARDS) and severe respiratory failure of type 1 resulted in three outcomes: fatality, recovery with lung fibrosis of different intensity or full recovery without pathological consequences for lungs. All patients with ARDS were oxygenated with the help of mechanical ventilators or extracorporeal membrane oxygenation (ECMO) apparatuses in intensive care units. The sum of column values in any of the four quintuplets is 100%.

**Figure 7** presents more detailed clinical picture and outcomes of SARS-CoV-2-related pneumonias. Of 1204 cases, there were 89 deaths (in-group mortality 7.39%). The detailed data are provided in Table S1 in the **Online Supplementary Document**.

**Figure 8:**
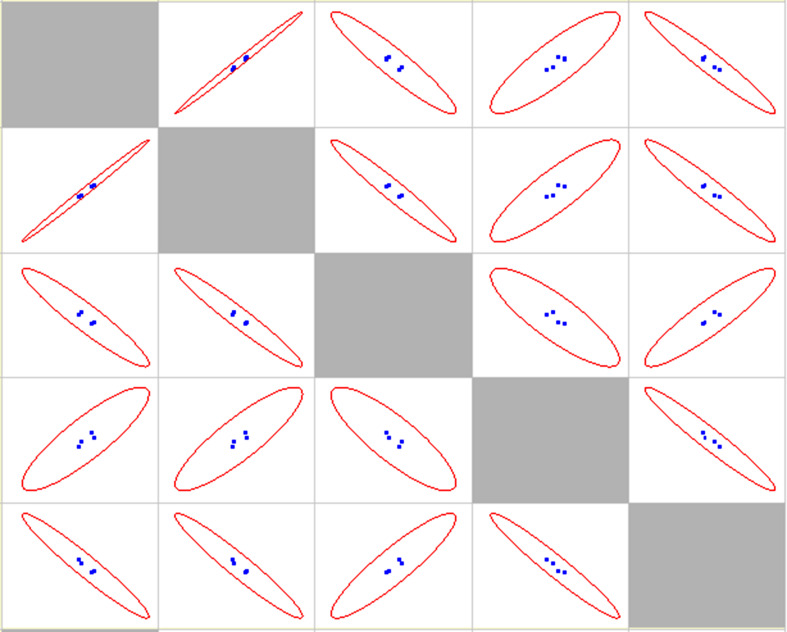
Scatter matrix for different cases of pneumonia aetiological agents presence other than SARS-CoV-2.

Correlation statistical analysis is provided in **Table 1**, scatter matrix is shown in **Figure 8**.

**Table 1:** Pearson and Spearman correlation coefficients for different clinical course*

	ARDS + Fatality	ARDS + Lung fibrosis on recovery	ARDS + Full recovery	NIV oxygenation	No respiratory failure
ARDS + Fatality	1	**0.9959** (1)	-0.9417 (-0.6)	0.8695 (0.8)	**-0.9656** (-0.8)
ARDS + Lung fibrosis on recovery	*P* = 0.004 (–)	1	**-0.9684** (-0.6)	0.8718 (0.8)	**-0.9612** (-0.8)
ARDS + Full recovery	*P* = 0.058 (*P* = 0.4)	*P* = 0.032 (*P* = 0.4)	1	-0.8462 (0.8)	0.9111 (-0.8)
NIV oxygenation	*P* = 0.130 (*P* = 0.2)	*P* = 0.128 (*P* = 0.2)	*P* = 0.154 (*P* = 0.2)	1	**-0.9667** (-1)
No respiratory failure	*P* = 0.034 (*P* = 0.2)	*P* = 0.039 (*P* = 0.2)	*P* = 0.089 (*P* = 0.2)	*P* = 0.033 (*–*)	1

ARDS – acute respiratory distress syndrome, NIV – non-invasive ventilation

*Spearman coefficients and corresponding significance levels are given in parentheses. Very strong correlations and anti-correlations (the module of Pearson coefficient is more than 0.95) are highlighted in bold.

**Figure 9:**
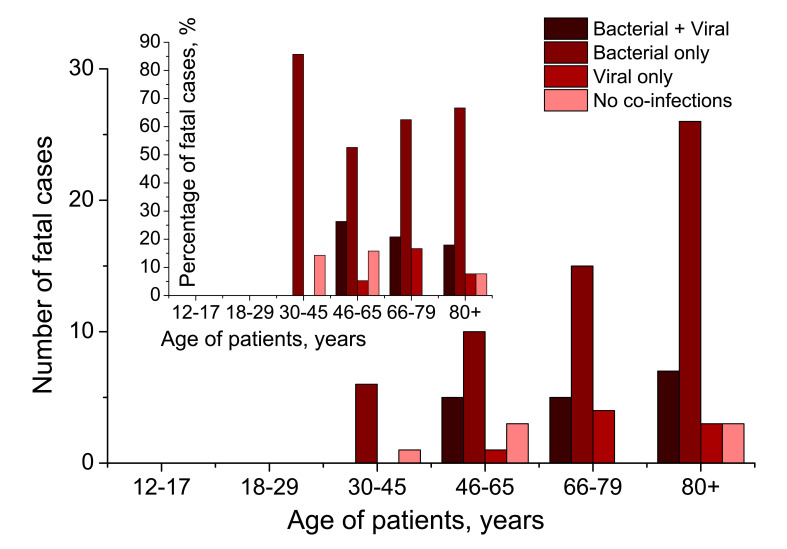
Age breakdown of mortality of pneumonia patients tested positive for SARS-CoV-2 with respect to different bacterial and/or viral co-infections. The absolute number of cases is provided in the main graph, and percentage is provided in the inset. The sum of column values in any of the four quadruplets in the inset is 100%.

Several tendencies may be observed in **Figure 7****.** The ratio of patients with most favourable clinical course of pneumonia, which did not require any oxygen ventilation (dark green columns), rises with going from the left to the right in the graph, from most complicated cases to no co-infection cases. The proportion of those patients, who were provided NIV oxygenation (light green columns), decreases from complicated to uncomplicated pneumonias. ARDS with successful recovery without any signs of lung fibrosis (violet columns) increases. The part of patients with more or less pronounced lung fibrosis after their recovery from pneumonia (brown columns), sharply falls for the cases non-complicated with secondary bacterial pneumonias. Finally, the overwhelming majority of fatalities (black columns) were of patients with secondary bacterial pneumonias (two left quintuplets). For viral-bacterial-complicated cases, there were 17 deaths of 111 patients (15.32%) and for bacterial-complicated cases, 57 deaths of 322 patients (17.70%). Therefore, the proportion of mortality in the group of bacterial-complicated pneumonias is twice as large in comparison with mortality in broad samplings of SARS-CoV-2-related pneumonias (8.16% in Set 1 and 7.39% in Set 2). For SARS-CoV-2-related pneumonias uncomplicated by secondary bacterial infections, mortality is strikingly lower (3.94% for viral co-infections and 1.23% for no co-infection cases). In comparison with secondary bacterial pneumonias, viral co-infections in addition to SARS-CoV-2 have much less impact on clinical course and outcome of pneumonia patients. **Figure 9** further elucidates the age breakdown of in-group mortality in Set 2. The detailed data are provided in Table S2 in the **Online Supplementary Document**.

**Figure 10:**
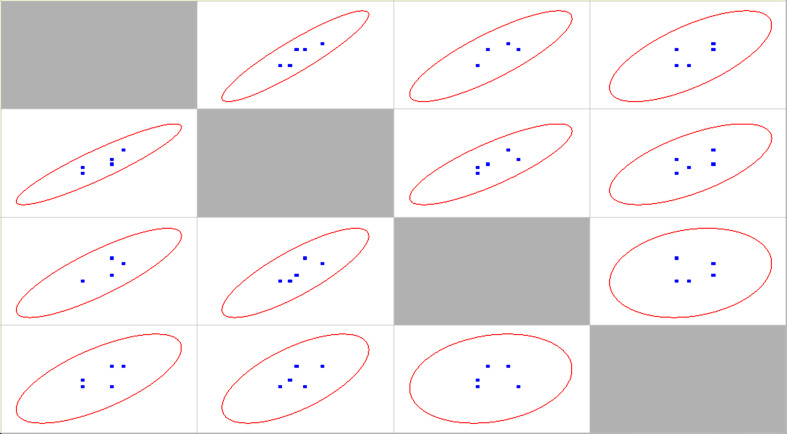
Scatter matrix for different age groups. 12-17 and 18-29 age groups are represented by one point, as they are statistically identical.

Correlation statistical analysis is provided in **Table 2**, scatter matrix in **Figure 10**.

**Table 2:** Pearson and Spearman correlation coefficients for different cases of aetiological agents presence other than SARS-CoV-2*

	Bacterial + viral	Bacterial	Viral	No co-infections
Bacterial + viral	1	**0.9168 (0.9393)**	0.8358 (0.8854)	(0.6889) (0.6167)
Bacterial	*P* = 0.010 (*P* = 0.005)	1	0.8260 (0.8933)	0.6483 (0.5949)
Viral	*P* = 0.038 (*P* = 0.019)	*P* = 0.043 (*P* = 0.017)	1	0.2069 (0.2623)
No co-infections	*P* = 0.130 (*P* = 0.192)	*P* = 0.164 (*P* = 0.213)	*P* = 0.694 (*P* = 0.616)	1

*Spearman coefficients and corresponding significance levels are given in parentheses. Very strong correlation (the module of Pearson/Spearman coefficient is more than 0.90) is highlighted in bold.

The absolute number of fatal cases rises with age (**Figure 9**, main graph), with bacterial-complicated pneumonias account for the largest amount of deaths of SARS-CoV-2-related pneumonia. However, the percentage of fatalities in different scenarios of presence/absence of additional co-agents in SARS-CoV-2 cases (**Figure 9**, inset), remains comparable for every age group. Indeed, we see that for groups 46-65, 66-79 and 80+ years, the proportions of deaths with secondary bacterial pneumonia, are very high, 78.95%, 83.33% and 84.62% of all deaths related to pneumonia initially caused by SARS-CoV-2, and these proportions are comparable for the three cohorts. Only seven deaths were registered for cohort 30-45 years, with mortality related to secondary bacterial infection, being 85.71% of all deaths observed for this cohort.

### Population mortality estimations of SARS-CoV-2-related pneumonia

**Figure 11:**
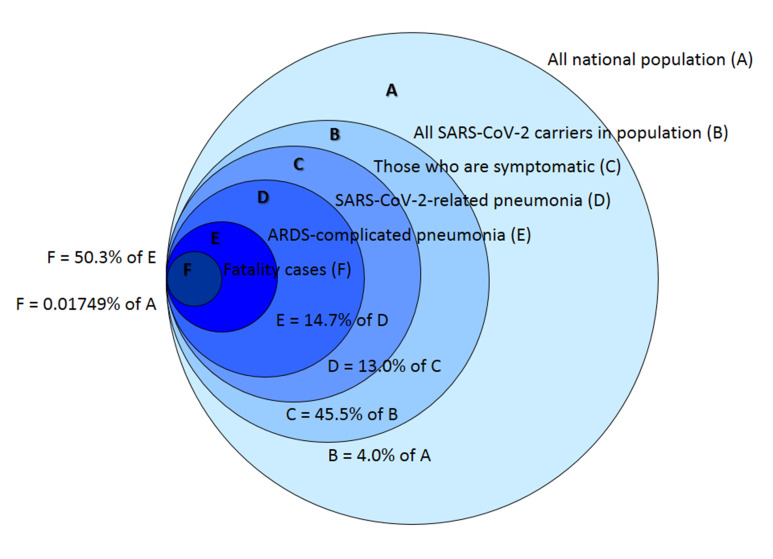
Different sets of people affected by SARS-CoV-2 in Russian population. Circles area proportions do not reflect with percentages of sets A-F. Current population infection rate B/A = 0.04 (official data on population mass screening) [38]. Ratio of symptomatic patients to all carriers C/B = 0.455 (our unpublished data on large sampling symptomatic study). Percentage of pneumonia COVID-19 patients in symptomatic cohort D/C = 0.13 (our unpublished data on large sampling symptomatic study). Ratio of ARDS complications in SARS-CoV-2 related pneumonia E/D = 0.147 (determined in the current study, on analysing Set 2). Mortality in cohort E F/E = 0.503 (determined in the current study, on analysing Set 2).

Calculation of mortality associated with COVID-19 in different cohorts has additional importance for estimating the national population mortality, if SARS-CoV-2 spread is not stopped and no vaccine is created in the nearest 2 years. Using our unpublished data and Russian official informational resources, we may evaluate the global mortality of COVID-19-related pneumonias in Russia as some 25 500 people on a scale of the nearest two years, or 12 250 people a year (**Figure 11**).

The two-year period is chosen as a mean time of spreading a novel respiratory virus around the globe. Such time interval was observed for swine flu H1N1 pandemic of 2009-2010 and other human coronaviruses (HCoV-HKU1, HCoV-OC43, HCoV-229E, and HCoV-NL63) epidemics that broke out during the last decades [39-41].

In 2010-2015, in Russia around 33 000-40 000 persons died of different bacterial, mycoplasma, viral and other types of pneumonia annually, in 2016 nearly 31 000 people, in 2017 around 26 000 people, and in 2018-2019 around 25 000 people (Rosstat official data) [29]. Therefore, annual mortality of COVID-19-related pneumonia, is substantial on the background of no-COVID pneumonia case fatality. Though it is twice or even thrice less than annual mortality due to pneumonias of different aetiology observed during the last decade, it may boost the overall lethality brought about by respiratory diseases in Russia within nearest 1-2 years appreciably, especially in risk groups of population, if no measures have been taken to eliminate nosocomial secondary pneumonia in COVID-19 cases. After these two years, there are reasons to believe that SARS-CoV-2 will transform to a seasonal viral agent in the ARI viruses group, as it had been with four other human coronaviruses [42]. Then mortality due to SARS-CoV-2 related pneumonia may drastically fall because of human population adaptation. A vaccine creation may facilitate and expedite this process.

However, even in the current situation of a vaccine absence, we may substantively reduce the Russian fatality number 25 500 persons to at least twice lesser value, by uncompromising combating hospital-acquired secondary pneumonia in COVID-19 pneumonia cases. Community-acquired pneumonias are difficult to control and manage, but nosocomial cases of secondary bacterial infections may be treated much more effectively and their overall quantity may be reduced, if a proper clinical algorithm has been elaborated and strictly followed.

### COVID-19 population mortality controversy in Russia

**Figure 12:**
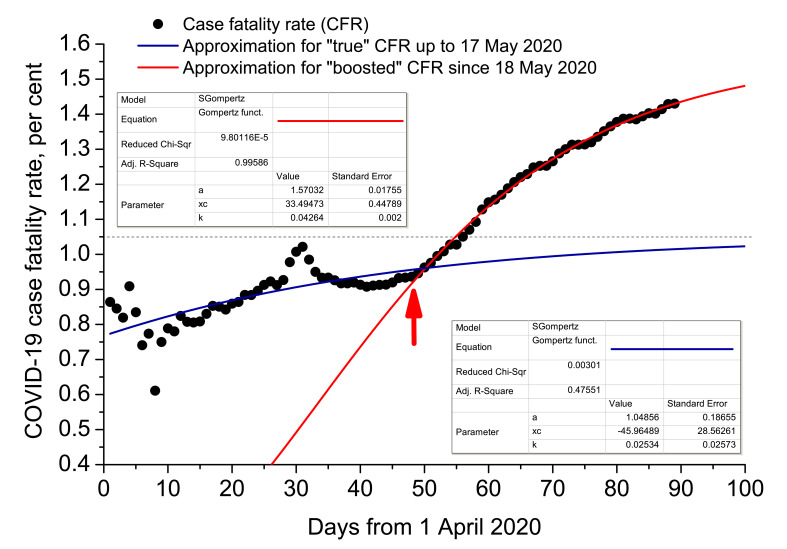
Change of protocols in calculating COVID-19-related mortality in Russia on 18 May 2020. Red arrow points at the date of change. Case fatality rate (CFR) = total deaths (accumulated) / total positive cases (accumulated). Official Russian statistics are taken into account [38]. Calculated regression factors, coefficients and their standard errors are presented in the insets.

For some reason, Moscow mayor office and Moscow health care department changed protocols of calculating COVID-19 case fatality rate since 18 May 2020, that may be corroborated by the announcement of Moscow health care department [43]. Till 17 May 2020, a death was ascribed to SARS-CoV-2 only if the pathogen was detected (pre- or post-mortem) and clear clinical proofs were present that it was COVID-19 that caused the fatality. Such an approach bounded COVID-19 case fatality rate in Russia at a level of nearly 0.9%. The new protocol prescribed to ascribe a death to SARS-CoV-2 without clear clinical evidence, post-mortem analysis and even the presence of RT-PCR positive test, if symptomatic manifestations were similar with those that are usually brought about by COVID-19. Since the proportion of COVID-19-related mortality of Moscow is around 95% in the whole of Russia, the protocol change boosted the SARS-CoV-2 observed mortality considerably (**Figure 12**).

Statistical regression analysis was carried out, taking into account that observed mortality due to a novel pathogen to which population has not adapted yet, may be described by Gompertz sigmoidal function very effectively [44,45]:


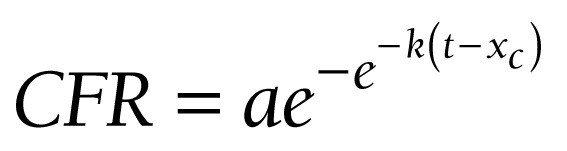


where CFR is case fatality rate, *a* is its upper achievable limit, *x_c_* is the time inflection point, and *k* is the slope coefficient. The analysis demonstrates that the aforesaid change of protocol artificially augmented COVID-19 CFR, making it 1.5 times higher (1.57 vs 1.05). The article was completed on 28 June 2020. Official mortality was 9073 humans for that day [38]. It means that, subtracting all non-COVID-19-related pneumonia mortality cases, we would receive 9073/1.5 ≈ 6050 people.

## CONCLUSIONS

COVID-19 pandemic is a global challenge, defiance to all of us and hazard to stability of health care systems in almost any state. It was already noted at the very beginning of SARS-CoV-2 pandemic in China and even before, that in the truly global society in which we live now, even low-pathogenic infection that was not contained at its nidus and instead spread across the planet, represents considerable hazard to sustainability in every corner of the world, from epidemiological and medical sustainability to economic and political sustainability [46-50]. Russian positive and negative experience in hospital treatment of COVID-19 pneumonia may be taken into consideration in medical care of other countries.

In the current study, we sustained our hypothesis that secondary bacterial pneumonia has been the main cause of lethality associated with COVID-19 in Russia in March-May 2020. SARS-CoV-2 is far not so hazardous in terms of contagiousness and case fatality as Ebola, Nipah or highly pathogenic avian flu H5N1 viruses, which have recently caused dramatic local outbreaks in Asia and Africa [51-54]. The current “true”, ie, recalculated CFR of COVID-19 is some 1.0% in Russia that is similar with Influenza A fatality. However, SARS-CoV-2 demonstrated its dangerousness in higher mortality in the group of patients with pneumonia caused by it (7.3%-8.2%) and substantially higher mortality in the group of patients with secondary bacterial pneumonia (15.3%-17.7%). Additionally, SARS-CoV-2 caused very unfavourable clinical course and outcome in cases of secondary bacterial pneumonia. Investigating possible reasons for this may be a matter of concern of another research. The proportion of secondary bacterial pneumonia in COVID-19-related pneumonia cases in Russia, was considerable in March-May 2020, around a third of all SARS-CoV-2 infected persons with pneumonia.

We found that secondary bacterial pneumonia complications accounted for the overwhelming majority of deaths associated with COVID-19-related pneumonias. Another important lethal complication of COVID-19 disease may be pulmonary embolism (lung artery thrombosis) [55-58]. Excessively large amount of all deaths of pneumonia patients tested positive for SARS-CoV-2 (78%-86%), was related to ARDS and severe respiratory failure of type 1 caused by secondary bacterial pneumonias.

### Global medical care perspectives

A very dangerous observation consists in the fact that 55.20% of secondary bacterial pneumonia cases were hospital-acquired. In comparison, studying Influenza A related pneumonia cases in Set 1, we estimated the proportion of hospital-acquired secondary pneumonia as no more than 15% of all secondary bacterial pneumonia cases accompanying Influenza A viral pneumonias. A possible explanation may consist in the fact that human population has not yet adapted to a novel coronavirus, while Influenza A viruses of different subtypes (eg, H1N1, H3N2) have already become common seasonal agents with worldwide spread.

Consequently, in hospital treating the COVID-19 patients every effort has to be done to diminish the impact of secondary bacterial infections.

First of all, secondary bacterial complications need be diagnosed as soon as possible. Not only CT should be applied to detect overall lungs image, but detailed analysis for different Gram-positive, Gram-negative and atypical bacterial aetiological agents must be performed with interval of no more than two days, to detect a probable deterioration of a patient’s situation immediately. Different bacteria may attack weak, depressed and emaciated hospital patients in different specific times (see Table S3 in the **Online Supplementary Document**, and [59,60]).

Sputum or BAL intakes with the following cultural inoculation methods paired with biochemical detection, are the best options. Here two probable difficulties arise. First, not always a patient’s condition allows performing such intakes with two-day intervals. For children, aged persons and severely ill, emaciated and exhausted patients, it is difficult to receive sputum or BAL samples with 48-hour intervals. Second, for some bacteria the methods of inoculation on broth are very difficult to carry out in clinical microbiological practice due to special requirements, cost, and length of analysis. In such cases, antigen blood/urine testing as well as RT-PCR test for the whole genome or specific genes may be applied, though the sensitivity and specificity are lower for many bacteria. However, it is to remember that nothing can be compared with cultural techniques for bacterial pneumonia aetiological agents, as these procedures give an opportunity to study resistance to antimicrobial drugs along with the growth of a culture. After a bacterial agent has been grown on broth in sufficient amounts, its resistance to antimicrobial chemicals should be investigated, while ample allowance of different antimicrobial medicines should be preserved in any hospital. In Russia, medical care system faced critical shortages of broad-action and highly specific antibiotics in March-April 2020, even in several central COVID-19 infirmaries in Moscow and St Petersburg. We must avoid such situations in the future.

In cases where sputum/BAL intake cannot be performed with 48-hour intervals, we would suggest the following protocol schematics for detecting secondary bacterial pneumonia in the hospital treatment of COVID-19-related pneumonia patients. 1. Blood and urine samples are taken every 24 hours or when a patient’s condition has been deteriorated noticeably, and analysed for antigens/antibodies to known bacterial agents. Nasopharyngeal or oropharyngeal swabs should be also taken on a 24-hour basis for PCR testing for DNA analysis (full genome or specific genes of given bacteria). Blood/urine investigation should be repeated twice, to avoid false-negative results due to insufficient sensitivity of many antigen/antibodies detection techniques. PCR analysis of a given sample should be repeated twice due to a risk of false-negative results due to a mistake of a technician, as in molecular biological testing this risk is considerable. 2. If there is a positive result for a secondary bacterial agent presence, sputum or BAL should be taken immediately for cultural analysis with further analysing the resistance to a panel of antimicrobial drugs. Blood, urine and swab intakes should be also immediately repeated. 3. If the second testing for antigen/antibodies and/or molecular biology give another positive result for a bacterial agent in question, broad standard treatment of the bacterial infection should be started at once by conventional antibiotics that are usually used in such cases. 4. Once the inoculation and resistance results are ready, a decision should be made whether the treatment by the antibiotic chosen may be prolonged or it is ineffective. 5. If it is ineffective, then a switch to another treatment should be made, based on the resistance test results. 6. Once a bacterial agent has been identified and corresponding antimicrobial treatment has commenced, sputum/BAL intakes should be carried out every 7 days, while blood/urine and swab intakes may be repeated on a semi-week basis, to check clinical picture and detect a new possible hospital-acquired infection, especially for children, aged people and immunosuppressed patients.

Among bacterial aetiological agents common for hospital-acquired pneumonia cases, there are many bacteria exceptionally resistant to treatment with broad-spectrum antibiotics. As we have seen, such bacteria, eg, *Pseudomonas aeruginosa, Staphylococcus aureus*, *Klebsiella pneumoniae, Chlamydophila pneumoniae, Moraxella catarrhalis, Legionella pneumophila,* and some other may dramatically exacerbate COVID-19 clinical course and undermine chances of a positive outcome. Analysis of Set 1 (**Figure 2**) demonstrates that mortality due to pneumonia caused by *Klebsiella, Chlamydophila, Moraxella* and *Legionella* is approaching 20 per cent, ie, exceeds mortality associated with *Streptococcus pneumoniae*, almost 10-fold. Although in our research, in Set 1 there were no fatalities associated with simultaneous presence of SARS-CoV-2 and *Pseudomonas aeruginosa*, the mortality rate of *Pseudomonas*-caused pneumonias can be also very high [60]. All these bacteria are mainly opportunistic infections that strike people with immunosuppressed status. Besides, all of them are enormously persistent, showing exceptional multidrug resistance to antibiotics. Eg, *Klebsiella* species often produce carbapenemases that hydrolyse beta-lactams [61-64]. Carbapenems are usually the medications of the last resort for treating highly resistant Gram-negative pneumonia aetiological agents [61]. Therefore, cases of SARS-CoV-2 pneumonia complicated by *Klebsiella* infection may leave scanty chances of the positive prognosis for a patient, if appropriate diagnostics, check for resistance potential to antibiotics and relevant choice of medications have not been done at the very beginning of the secondary pneumonia disease. Serious cases of complicated secondary pneumonia may probably require combined therapy by a “cocktail” of highly specific antibiotics and bacteriophages [65-71].

The risk of community-acquired and hospital-acquired secondary pneumonias and their further complications, should be treated very seriously in combating SARS-CoV-2. Elimination or at least considerable reduction of secondary bacterial pneumonia cases in hospitals would be a proper, relevant and necessary step towards abatement of the entire COVID-19 threat to population.

## Additional material

Online Supplementary Document
