# Supplementary material for: SARS-CoV-2-related pneumonia cases in pneumonia picture in Russia in March-May 2020: Secondary bacterial pneumonia and viral co-infections
Source: J Glob Health. 2020 Aug 18;10(2):020504. doi: 10.7189/jogh.10.-020504 (PMC7568231; doi:10.7189/jogh.10.-020504)
Supplement: Online Supplementary Document [file jogh-10-020504-s001.pdf]

Supplementary Material

Giddings peak function (introduced by J. Calvin Giddings in 1972):

$P_{viral\ co-infect.} = y_0 + \frac{A}{w}\sqrt{\frac{x_c}{t}}x^{-\frac{x_c+t}{w}} I_1\left(\frac{2\sqrt{x_c t}}{w}\right)$, where I_1 may be represented as a series: $I_1(x) = \sum_{k=0}^{\infty}\frac{1}{k!(k+1)!}\left(\frac{x}{2}\right)^{2k+1}$.

Table S1. Absolute number of SARS-CoV-2 pneumonia patients in Set 2 (1,204 patients) in different cohorts corresponding with different disease clinical course and outcome.

Aetiological agents	ARDS, Fatality	ARDS, Lung fibrosis on recovery	ARDS, full recovery	Oxygenation through NIV	No respiratory failure observed	Total
Bacterial + Viral together	17	6	2	44	42	**111**
Bacterial only	57	19	8	104	134	**322**
Viral only	8	1	15	53	126	**203**
No co-infections	7	0	37	101	423	**568**
**Total**	**89**	**26**	**62**	**302**	**725**	**1204**
**Percentage of the whole Set 2**	7.39%	2.16%	5.15%	25.08%	60.22%	

ARDS – acute respiratory distress syndrome
NIV – non-invasive ventilation

Table S2. Absolute number of deaths related to SARS-CoV-2 pneumonia in Set 2, within different age cohorts.

Age range	Bacterial + Viral together	Bacterial only	Viral only	No co-infections	Total
12-17	0	0	0	0	**0**
18-29	0	0	0	0	**0**
30-45	0	6	0	1	**7**
46-65	5	10	1	3	**19**
66-79	5	15	4	0	**24**
80+	7	26	3	3	**39**
**Total**	**17**	**57**	**8**	**7**	**89**
**Percentage**	19.10%	64.04%	8.99%	7.87%	

Table S3. Number of community-acquired and hospital-acquired bacterial and viral secondary pneumonia cases in Set 2.

Aetiological agent	Community-acquired	Hospital-acquired (nosocomial)	Total	Percentage of hospital-acquired pneumonia	Mean time of detection after the treatment has been initiated, for hospital-acquired pneumonia cases (CI = 95%, *p* = 0.05), days
Streptococcus pneumoniae	93	18	**111**	16.22%	2.6 ± 0.9
Staphylococcus aureus	26	66	**92**	71.74%	4.2 ± 1.5
Haemophilus influenzae	18	70	**88**	79.55%	5.1 ± 2.3
Escherichia coli	46 (only for 12-17 age cohort)	5	**51**	9.80%	6.6 ± 4.1
Mycoplasma pneumoniae	7	33	**41**	80.49%	7.4 ± 1.7
Chlamydophila pneumoniae	2	36	**38**	94.74%	8.1 ± 2.2
Klebsiella pneumoniae	1	11	**12**	91.67%	7.2 ± 2.8
**Orthopneumovirus HRSV**	86	16	**102**	15.69%	7.7 ± 2.5
**Rhinoviruses A, B, C**	88	3	**91**	3.30%	2.9, unreliable CI
**Adenoviruses B, C, E**	14	27	**41**	65.85%	6.8 ± 1.3
**Metapneumovirus subtypes A, B**	19	15	**34**	44.12%	7.1 ± 2.9
**Parainfluenza viruses types 1,2,3 4**	12	18	**30**	60.0%	4.4 ± 1.7
**Cytomegalovirus HHV-5**	16 (only for 12-17 age cohort)	0	**16**	0	
**Total**	428	318	**746**		
**Percentage**	57.37%	42.63%*			

* 55.20% in bacterial subgroup and 25.16% in viral subgroup
